# The Role of Innate Immunity and Bioactive Lipid Mediators in COVID-19 and Influenza

**DOI:** 10.3389/fphys.2021.688946

**Published:** 2021-07-22

**Authors:** Sabina Sahanic, Judith Löffler-Ragg, Piotr Tymoszuk, Richard Hilbe, Egon Demetz, Rebecca K Masanetz, Markus Theurl, Johannes Holfeld, Can Gollmann-Tepeköylü, Alexandar Tzankov, Guenter Weiss, Martin Giera, Ivan Tancevski

**Affiliations:** ^1^Department of Internal Medicine II, Medical University of Innsbruck, Innsbruck, Austria; ^2^Department of Internal Medicine III, Medical University of Innsbruck, Innsbruck, Austria; ^3^Department of Cardiac Surgery, Medical University of Innsbruck, Innsbruck, Austria; ^4^Institute of Medical Genetics and Pathology, University Hospital Basel, Basel, Switzerland; ^5^Center for Proteomics and Metabolomics, Leiden University Medical Center, Leiden, Netherlands

**Keywords:** innate immunity, COVID-19, lipid mediator, metabololipidomics, influenza virus, macrophages and neutrophils

## Abstract

In this review, we discuss spatiotemporal kinetics and inflammatory signatures of innate immune cells specifically found in response to SARS-CoV-2 compared to influenza virus infection. Importantly, we cover the current understanding on the mechanisms by which SARS-CoV-2 may fail to engage a coordinated type I response and instead may lead to exaggerated inflammation and death. This knowledge is central for the understanding of available data on specialized pro-resolving lipid mediators in severe SARS-CoV-2 infection pointing toward inhibited E-series resolvin synthesis in severe cases. By investigating a publicly available RNA-seq database of bronchoalveolar lavage cells from patients affected by COVID-19, we moreover offer insights into the regulation of key enzymes involved in lipid mediator synthesis, critically complementing the current knowledge about the mediator lipidome in severely affected patients. This review finally discusses different potential approaches to sustain the synthesis of 3-PUFA-derived pro-resolving lipid mediators, including resolvins and lipoxins, which may critically aid in the prevention of acute lung injury and death from COVID-19.

## Introduction: General Concepts of SARS-CoV-2 Infection and Associated Inflammation

COVID-19, the infectious disease caused by the novel coronavirus SARS-CoV-2, currently represents a worldwide medical, economic, social, and political challenge (Mahase, 2020). SARS-CoV-2 was discovered in Wuhan, China, in December 2019 and has rapidly spread all over the world. In January 2020, the World Health Organization has declared a “Public Health Event of International Concern” and since March 11, 2020, COVID-19 has been characterized as a worldwide pandemic. Overall mortality rates are highly variable and range from 0.5 to 7%, very much depending on the stringency of testing in a given region, age, comorbidities of the patient, and access to medical treatment. When focusing on hospitalized patients, 20–40% were admitted to the intensive care unit (ICU) due to severe lung pathology needing ventilatory assists. Of these, >50% required invasive mechanical ventilation (Karagiannidis et al., 2021) and 15–22% of these patients were reported to die in hospital, highlighting the potential threat to public health (Huang et al., 2020).

As of March 2021, the number of global deaths due to COVID-19 exceeds already 2,700,000 people (Johns Hopkins Coronavirus Resource Center, 2021). In the 2002–2003 SARS-CoV-1 outbreak, the clinical course was characterized by fever, cough, myalgia, and other systemic symptoms that generally improved after a few days, followed by a second phase with recurrence of fever and severe pneumonia, associated with a high case fatality rate of 11% (Peiris et al., 2003; Hui et al., 2005). SARS-CoV-1 and SARS-CoV-2 are phylogenetically closely related and cause a similar biphasic clinical course, but they are phylogenetically closely related (Walls et al., 2020). From a structural point of view, both coronaviridae share high homology for their transmembrane spike (S) glycoprotein, a viral surface protein crucial for entry into host cells and for initiation of immune response (Jaimes et al., 2020; Monteil et al., 2020; Walls et al., 2020). Specifically, both SARS-CoV interact directly with angiotensin-converting enzyme 2 (ACE2) *via* the S protein to enter alveolar cells and are believed to induce acute respiratory distress syndrome (ARDS) through ACE2 downregulation and shedding (Imai et al., 2005, 2008; Kuba et al., 2005, 2006; Blanco-Melo et al., 2020; Fu et al., 2020). Intrapulmonary loss of ACE2 leads to accumulation of angiotensin II, which appears to play a central role in the release of inflammatory cytokines, resulting in the activation of the IL-6 amplifier, which describes stimulation of the NF-κB and the JAK-STAT3 pathways resulting in inflammatory cytokine formation (Imai et al., 2005, 2008; Kuba et al., 2005, 2006; Blanco-Melo et al., 2020; Fu et al., 2020; Moore and June, 2020).

SARS-CoV-2 patients suffering from a complicated course of infection either fail to exert a robust, interferon (IFN)-mediated anti-viral response in the early phase of infection and present with an overwhelming immune activation termed as “cytokine storm” (Blanco-Melo et al., 2020; Fu et al., 2020). The latter is defined by increased levels of circulating cytokines accompanied by systemic and pulmonary immune cell activation in a similar setting as described in subjects suffering from ARDS or sepsis (Wilson et al., 2020). Importantly, patients with severe COVID-19 show loss-of-function variants in Toll-like receptor (TLR)- and IFN-dependent genes, or neutralizing antibodies to type I IFN (*α* and *ω*; Bastard et al., 2020; Zhang et al., 2020a). In addition, there are marked variance and temporal changes in the IFN gene signature during the course of COVID-19, possibly driving immunopathology (Nienhold et al., 2020).

Since its appearance in late 2019, COVID-19 has been repeatedly compared to other viral infections and among others mainly to influenza. From an epidemiological perspective, it seems reasonable to compare seasonal flu with COVID-19, given that they are respiratory diseases with similar modes of transmission. However, patients affected by COVID-19 exert strikingly different predisposing comorbidities and a more severe clinical course with higher morbidity and mortality rates as compared to seasonal influenza (Piroth et al., 2021). Patients affected by COVID-19 are more frequently obese or overweight, and show higher incidences of diabetes, hypertension, and dyslipidemia compared to patients with severe influenza (Piroth et al., 2021). The most obvious reason for the marked differences observed in epidemiology and fatality rates relies on the fact that SARS-CoV-2 engages immunological and thrombo-inflammatory circuits (Bösmüller et al., 2021) that differ from the well-known IFN-based response to influenza virus. Therefore, this review will initially focus on innate immunity in influenza and in SARS-CoV-2 infection comparing the respective host immune signatures, and subsequently depict recent findings on the mediator lipidome in COVID-19.

## Innate Immunity in Influenza Virus Infection

Despite both bearing the potential of causing a severe infection of the lung, influenza and SARS-CoV-2 elicit several pathways of innate immunity that differ in many aspects. The main immunological differences between the “classical” immune response to influenza virus and the aberrant, “SIRS-like” response to SARS-CoV-2 important for this review will be outlined as follows:

Immunological responses to influenza are mainly driven by coordinated type I and III IFN release following TLR3, TLR7, and TLR8 activation. Besides TLR3 and TLR7/8, RIG-I-like receptors as well as nucleotide-binding oligomerization domain (NOD)-like receptors (NLRs) are known to initiate the immune response to influenza virus. Whereas endosomal TLR3 detects double-stranded RNA (dsRNA), TLR7 and TLR8 sense single-stranded RNA (ssRNA); RIG-I specifically recognizes 5'-triphosphate RNA; and NLRs may directly recognize viral products, leading to the formation of inflammasomes (Biondo et al., 2019). RIG-I and NLRs have been comprehensively reviewed elsewhere (Root-Bernstein, 2021) and will not be covered in more detail here.

TLR3 differs from other TLRs in using TRIF (TIR domain-containing adaptor protein-inducing IFN-*β*) as signal adaptor, leading to synthesis of type I IFN mainly through the activation of NF-κB and IRF3 (Kawai and Akira, 2010; Ullah et al., 2016). In addition, dsRNA released from damaged cells may also activate TLR3 in resident or recruited macrophages in the lungs, which actively phagocytose dying and apoptotic cells (Schulz et al., 2005; Gosu et al., 2019). TLR3 appears to play an important role in influenza A virus (or seasonal influenza, H1N1)-induced innate host defense: Mice deficient in TLR3 had an unexpected survival advantage in the H1N1 infection model, despite a higher viral load in the lungs (Le Goffic et al., 2006). Mechanistically, TLR3-induced secretion of type I IFNs promotes the expression of the so-called IFN-stimulated genes (ISGs) within infected cells, including the serine–threonine kinase protein kinase R, IFITM3, and the myxovirus resistance protein 1 (MX1; Iwasaki and Pillai, 2014; Schoggins, 2014). ISGs inhibit viral entry into the cytosol as well as virus replication in the different cellular compartments of the lung. Moreover, type I IFNs potently activate natural killer (NK) cells, which kill virus-infected cells. Besides NK cells, also neutrophils play an important role in response to acute H1N1 infection.

The contribution of neutrophils to the pathology conferred by influenza is exemplified by the 1918 pandemic virus, which induces a massive neutrophil recruitment to the lungs (Kobasa et al., 2004). Neutrophils may be protective at low virus titers by ingesting apoptotic cells, whereas they may further destroy the lung parenchyma when recruited at high numbers. Specifically, the release of reactive oxygen species and neutrophil extracellular traps may aid in the development of ARDS (Tate et al., 2009; Narasaraju et al., 2011).

Finally, alveolar macrophages appear to play a pivotal role in the host defense against influenza. Alveolar macrophages, together with epithelial and dendritic cells, were found to produce anti-viral type I IFNs, but also several proinflammatory cytokines and chemokines capable of attracting neutrophils and monocytes (De Jong et al., 2006; Jayasekera et al., 2006; Narasaraju et al., 2011). Recruited monocytes differentiate into inflammatory macrophages, which greatly amplify cytokine production. Both macrophages and neutrophils can ingest H1N1-infected cells, particularly when they are damaged or apoptotic, thereby promoting viral clearance and elimination of cell debris (Tumpey et al., 2005; Watanabe et al., 2005).

## Innate Immunity in SARS-CoV-2 Infection

**Figure 1:**
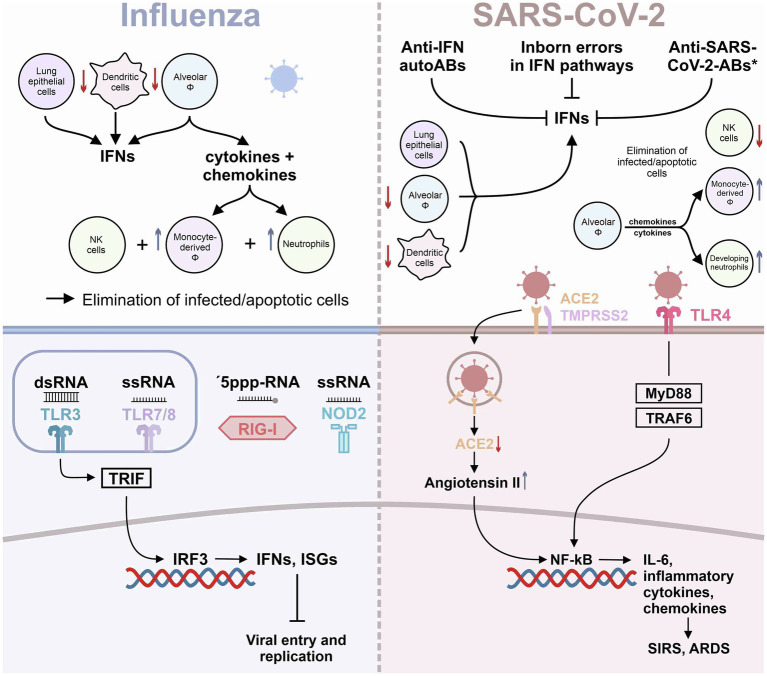
Innate immunity in influenza and SARS-CoV-2 infection. The graph summarizes the current knowledge on innate immunity responses in influenza virus and in SARS-CoV-2 infection of the lung. ^*^refers to anti-SARS-Cov-2 antibodies engaging with CD32B Fc receptors (Combes et al., 2021).

In contrast to influenza virus, SARS-CoV-2 was found to activate TLR4 (Figure 1) and TLR4-related pathways through binding of its spike protein in human and murine macrophages, resulting in IL-6-mediated hyperinflammation (Shirato and Kizaki, 2021). TLR4 activation by SARS-CoV-2 spike subunit S1 can be suppressed by selective inhibitors of NF-κB and JNK pathways (Shirato and Kizaki, 2021). Interestingly, *in silico* studies had predicted TLR4 to recognize molecular patterns of SARS-CoV-2 (Choudhury and Mukherjee, 2020). Direct activation of the TLR4 may switch the anti-viral response of a cell from a response otherwise dominated by type I IFNs to the release of mainly pro-inflammatory mediators, explaining at least in part the hyperinflammation associated with severe COVID-19. In addition, type I IFN response may further be blunted by changes in the Fc component of SARS-CoV-2-directed antibodies, as a recent study suggested (Combes et al., 2021). During the course of a disease, the characteristics of newly produced antibodies may fine-tune the immune response. One aspect of these changes is an alteration in the antibody Fc component that determines which Fc receptors will be engaged (Gentili and Hacohen, 2021). In this regard, engagement with the Fc receptors CD64, CD16, and CD32 can determine how the immune system combats viral infections. Using immune cells from healthy donors exposed to IFN-*α* and plasma from patients with severe COVID-19, Combes et al. individually blocked CD64, CD16, and CD32 Fc receptors and found that CD32 blockade enabled the expression of IFN-regulated genes (Combes et al., 2021; Gentili and Hacohen, 2021). Importantly, the CD32 Fc receptor exists in the two forms, CD32A and CD32B, respectively. CD32A engagement activates the immune system, whereas CD32B dampens immune responses (Gentili and Hacohen, 2021). Combes and colleagues showed that the inhibition of IFN-regulated genes, including IFITM3 and MX1, in severe COVID-19 cases was due to CD32B engagement. These data indicate that patients with severe COVID-19 may develop antibodies that interact with CD32B Fc receptors and thereby blunt IFN-mediated host defense (Combes et al., 2021). Accordingly, a subset of ISG-expressing monocytes and neutrophils was identified only in blood samples of patients with moderate disease and was almost absent in patients with severe COVID-19 (Combes et al., 2021).

Corroborating a failure in IFN response in severe COVID-19 cases, Casanova and coworkers recently identified patients with severe COVID-19 that bear mutations in genes involved in the regulation of type I and III IFN immunity. Specifically, loss-of-function mutations were found in genes that govern TLR3- and IFN regulatory factor 7 (IRF7)-dependent type I IFN immunity to influenza virus (Zhang et al., 2020a). In addition, another study by this laboratory identified individuals with high titers of neutralizing autoantibodies against type I IFN-*α*2 and IFN-*ω* in about 10% of patients with severe COVID-19 pneumonia (Bastard et al., 2020). These autoantibodies were found neither in infected people who were asymptomatic nor in those with mild infection or in healthy individuals (Bastard et al., 2020).

Further evidence for a dysregulated type I IFN response in severe SARS-CoV-2 infection comes from studies in patients previously infected with phylogenetically closely related SARS-CoV-1 and MERS-CoV, where blunted IFN response was associated with severe pathology and disease (Cameron et al., 2007; Channappanavar et al., 2016; Kindler et al., 2016). Overall, patients with severe COVID-19 infection may present with typical features of macrophage activation syndrome which is partly resulting from overwhelming IFN*γ* formation (Webb et al., 2020) as reflected by higher levels of the IFN*γ*-inducible macrophage-derived biomarker neopterin in subjects with severe COVID-19 (Bellmann-Weiler et al., 2021).

## Comparison of Innate Immunity in Influenza and SARS-CoV-2 Infection

Altogether, mild-to-moderate SARS-CoV-2 infections appear to resolve at least in part due to an adequate anti-viral IFN-mediated response. In contrast, severe cases of COVID-19 not only fail to build up a robust type I IFN response in the initial phase but also show an uncontrolled hyperinflammatory response in the subsequent course of infection leading to multi-organ damage and death. Some reports moreover suggest that in severe COVID-19 patients, uncoordinated IFN response may further amplify TNF/IL-1*β*-centered hyperinflammatory signatures (Lee et al., 2020). This is *per se* not contradictory to the total absence of IFNs in patients born with loss-of-function mutations in type I IFN pathways. In the latter setting, patients will experience a delayed clearance of virus together with an exaggerated immune response further amplified by IFNs. On the other hand, in patients with inborn errors in IFN-related genes unrestrained viral replication will end up in an overwhelming infection leading to an exaggerated myeloid cell activation. The initial failure to mount an efficient anti-viral immune response is also supported by the finding of low lymphocyte and specifically CD4^+^ counts to be associated with higher cytokine levels and a more severe course of the infection (Zhang et al., 2020b).

Again emphasizing the difference between influenza and COVID-19, a recent report shows that in circulating leukocytes of COVID-19 patients, ISGs are expressed at a higher level than in healthy subjects but are more muted than seen with seasonal coronaviruses and much lower than seen in influenza infection (McClain et al., 2020). Shedding light on the impact of dysregulated immune responses on the recruitment of innate immune cells, Blanco-Melo et al. showed that COVID-19 patients exhibit elevated serum levels of pro-inflammatory cytokines: Increased levels of CXCL9 and CXCL16 may serve to attract NK cells, CCL8 and CCL2 to recruit monocytes and macrophages, and CXCL8 to recruit neutrophils (Blanco-Melo et al., 2020). Interestingly, using a comprehensive single-cell RNA sequencing approach, Blish and coworkers showed that during COVID-19 infection, several innate immune cell subsets are depleted, including *γ*δ T cells, plasmacytoid dendritic cells (pDCs), and NK cells (Wilk et al., 2020). Loss of pDCs and NK cells may hamper viral elimination first by absent IFN I signal amplification *via* IRF7 (Zhang et al., 2020a) and second by reduced elimination of virus-infected cells. Moreover, the authors showed a significant loss of anti-inflammatory CD16^+^ non-classical monocytes which are recognized as the first line of defense in recognition and clearance of pathogens (Narasimhan et al., 2019; Winkler et al., 2021).

Finally, a novel cell population annotated as “developing neutrophils” was significantly increased only in COVID-19 patients with ARDS, which seems to represent emergency granulopoiesis (Yvan-Charvet and Ng, 2019; Wilk et al., 2020). Single-cell RNA sequencing of immune cells retrieved from bronchoalveolar lavage fluid (BALF) indicated loss of alveolar macrophages in severe cases accompanied by recruitment of inflammatory monocytes and neutrophils, resulting in a highly proinflammatory microenvironment in the lung (Liao et al., 2020). Corroborating the results by Blish and coworkers (Wilk et al., 2020), the latter study described reduced pDCs and NK cell numbers also in BALF of severe COVID-19 cases, inferring a systemic depletion of these leukocyte subsets rather than intrapulmonary exhaustion (Liao et al., 2020). Similar to what was described in humans with severe COVID-19, macrophages were found to drive inflammation within the lungs also in African green monkeys subjected to SARS-CoV-2 infection. Monocyte-derived newly recruited rather than resident alveolar macrophages were found to clear infected cells and debris, aiding in the resolution of infection (Speranza et al., 2021). Loss of alveolar macrophages during SARS-CoV-2 infection was present also in K18-hACE2-transgenic mice, with intrapulmonary neutrophil and Ly6C^+^ monocyte [the murine equivalent to CD14^+^CD16^−^ classical monocytes in men (Ziegler-Heitbrock et al., 2010)] numbers increasing over 7 days post-infection (Winkler et al., 2020; The main mechanisms of inflammation in influenza as opposed to SARS-CoV-2 infection are summarized in Figure 1).

## Bioactive Lipid Mediators in Virus Infection, From Influenza Virus to SARS-CoV-2

With respect to alveolar inflammation, bioactive lipids are of highest significance. This is exemplified by the fact that eicosanoid metabolism has taken center stage as druggable axis in asthmatic disease. Activation of immune cells of myeloid and monocytic origin results in the release of arachidonic acid (AA) mediated by phospholipases (Demetz et al., 2014). Subsequent metabolism of AA with 5-lipoxygenase (5-LOX) as central enzyme results in the formation of leukotrienes, such as leukotriene B4 (LTB4) or the cysteinyl-leukotrienes as, for example, leukotriene D4 (Haeggström, 2018). Leukotrienes act *via* the BLT and cysteinyl leukotrienes (CysLT) receptors. LTB4 is a pivotal chemotactic agent for neutrophils in the initial phase of inflammation, which under physiological conditions is followed by a temporal switch in lipid mediators finally leading to resolution and tissue homeostasis (Spite et al., 2014). While maximal levels of LTB4 are reached as neutrophils infiltrate the infected lung, other eicosanoids, including the prostaglandins PGE2 and PGD2, lead to a lipid mediator class switch (Levy et al., 2001). This class switch initiates translational regulation of the enzymes required for the production of pro-resolving lipid mediators, including lipoxin A4 (LXA4; Spite et al., 2014) and inhibition of platelets (Braune et al., 2020). LXA4 serves as endogenous regulator of neutrophil trafficking, and its production is associated with cessation of neutrophil infiltration during the inflammatory response (Levy et al., 2001). The resolution phase of inflammation is characterized by the recruitment of monocytes to the injured tissue which then differentiate into macrophages and actively clear apoptotic cells (Spite et al., 2014). Besides LXA4, further so-called specialized pro-resolving lipid mediators (SPMs) are synthetized during this phase of inflammation, including resolvins, protectins, and maresins. SPMs blunt neutrophil infiltration, decrease pro-inflammatory mediator production, and stimulate macrophage-dependent uptake of apoptotic leukocytes as well of cell debris (Levy et al., 2001; Spite et al., 2014). Failure of such a tightly orchestrated resolution will end up in chronic inflammation and tissue damage.

Inflammatory stimuli of the lung may moreover lead to the production of CysLT, such as LTD4 mainly causing smooth muscle cell contraction in the respiratory tract (Gentile et al., 2003; Haeggström, 2018). This finding has led to the development of the CysLT 1 receptor antagonists (e.g., montelukast) as well as the 5-LOX inhibitor zileuton, highly useful drugs in the management of asthmatic disease (Wenzel and Kamada, 1996; Hon et al., 2014; Theron et al., 2014; Dahlin et al., 2016). A combination therapy has recently also been suggested for the use in patients infected with SARS-CoV-2 (Funk and Ardakani, 2020). Finally, eicosanoid metabolism is well known to play an important role in platelet activation which may be an additional link to the observed frequent thrombotic complications during SARS-CoV-2 infection (Gupta et al., 2020; Bösmüller et al., 2021). In turn, lipid and in particular eicosanoid metabolism deserve increased attention, as possible druggable pathways in SARS-CoV-2. For additional information, please refer to a recent overview of eicosanoid metabolism in SARS-CoV-2 (Hoxha, 2020).

Upon viral infection of the lung, SPMs appear to be involved in immunopathology, which include docosahexaenoic acid (DHA)-derived protectins and D-series resolvins (RvD1-RvD6), and the eicosapentaenoic acid (EPA)-derived E-series resolvins (Serhan et al., 2002, 2006, 2015; Duffield et al., 2006; Schwab et al., 2007; Serhan, 2007; Serhan and Petasis, 2011; Arita, 2012; Isobe et al., 2012; Imai, 2015; Libreros et al., 2021). In a systems biology approach, Imai and colleagues identified protectin D1 (PD1) to protect from lethal H5N1 influenza infection in mice by impairing virus replication *via* the RNA export machinery (Morita et al., 2013). Interestingly, by comparing PR8/H1N1 with the low-pathogenicity influenza strain X31/H3N2, Tam et al. showed that 5-LOX metabolites correlated with the pathogenic phase of infection, whereas 12/15-LOX metabolites were associated with the resolution phase (Tam et al., 2013).

In her review *Role of omega-3 PUFA-derived mediators, the protectins, in influenza virus infection*, Yumiko Imai concluded that despite their main limitation of a short half-life, omega-3-derived PUFA, including PD1 and stable analogs, may represent an attractive strategy to treat influenza infection (Imai, 2015).

Besides being implicated in the synthesis of lipoxins, 5-LOX main action lies in the production of omega-6 PUFA-derived leukotrienes and prostaglandins upon infection, driving leukocyte recruitment and activation, vasodilation, bronchoconstriction, and vasopermeability, as outlined above. Thus, increasing omega-3 PUFA and decreasing omega-6 PUFA levels may represent a possible mean to skew the immune response toward resolution of inflammation, which led to the conception of the *COVID-Omega-F* Trial with the aim of resolving the cytokine storm in COVID-19 patients by supplementation of hospitalized patients with high-dose omega-3 PUFA i.v. over 5 days (Arnardottir et al., 2021), ClinicalTrials.gov Identifier: NCT04647604, estimated study termination date April 30, 2021. However, the situation seems more complex than a simplified omega-3/omega-6 classification would reflect. For example, while prostaglandin E2 is frequently regarded as pro-inflammatory mediator, several recent studies have underlined its anti-inflammatory and tissue regenerative functions (FitzGerald, 2015; Duffin et al., 2016). Along these lines, a recent phase II trial by Haeberle et al. is testing the synthetic prostacyclin (PGI2) analog iloprost for the treatment of ARDS (Haeberle et al., 2020). Moreover, just recently, the omega-6 PUFA adrenic acid and its derivatives have been shown to exert anti-inflammatory properties (Brouwers et al., 2020). Additionally, linolenic acid has been reported as potential substrate for 15-LOX, producing trihydroxyoctadecenoic acids in eosinophils with potential anti-inflammatory/pro-resolving functions (Fuchs et al., 2020). Overall, cell-specific as well as spatiotemporal effects will ultimately sketch a detailed picture of SARS-CoV-2-related changes in lipid mediator biosynthesis; up to now, only a limited number of studies have addressed such changes, as outlined below.

Due to the striking differences in the response of the innate immune system, lipidomics data derived from lethal influenza infection may not directly be applicable to a severe infection with SARS-CoV-2. However, lipid mediators, including PD1, RvDs, and RvEs, exert strong anti-inflammatory activity. In this regard, supplementation with omega-3 PUFA showed controversial results in patients affected by ARDS. While IV emulsions with DHA and EPA were shown to be protective (Pontes-Arruda and Hirasawa, 2011), dietary supplementation with fishoil or with n-3 fatty acids, *γ*-linolenic acid, and antioxidants did not show a clear benefit in ARDS (Rice et al., 2011; Stapleton et al., 2011). On the other hand, independent meta-analyses indicated that supplementation with omega-3 PUFA in patients with ARDS would associate with improvements in the PaO2/FiO2 ratio, with a shorter ICU stay, a shorter duration of mechanical ventilation, and a trend toward reduced mortality (Dushianthan et al., 2019; Langlois et al., 2019; Arnardottir et al., 2021).

Up to date, little is known about changes in the mediator lipidome during acute SARS-CoV-2 infection. There is however one comprehensive analysis showing that serum from patients with a moderate and severe COVID-19 course displays specific differences in abundance of immune regulatory and pro-inflammatory lipid mediators (Schwarz et al., 2021). The authors show that moderate versus severe infections were characterized by unique lipidomic profiles. Of particular interest was the observation that specific pro-resolving mediators, including RvE3 and RvD4, were increased in serum from patients with moderate COVID-19 compared to subjects with severe disease. Moderate disease furthermore was associated with increased levels of PGs, particularly PGE2. In contrast, severe COVID-19 was associated with a trend to increased serum levels of D-series resolvins RvD1-3 and LXA4 (Schwarz et al., 2021). Bioactive lipid mediators are generated by sequential activity of different enzymes, namely, 5-LOX, 12-LOX, 15-LOX, COX, and cytochrome p450 (Cyp450). Grouping according to different enzymatic pathways showed that moderate disease was characterized by higher levels of lipid mediators that require COX and 12-LOX activity, whereas severe disease was characterized by lipid mediators that require activity of 5-LOX and Cyp450 (Schwarz et al., 2021). By mining a published single-cell RNA sequencing dataset in PBMCs from severe COVID-19 patients, the authors found increased *ALOX5* expression in CD14^+^ and CD16^+^ monocytes and in neutrophils reflecting emergency granulopoiesis (Schwarz et al., 2021).

**Figure 2:**
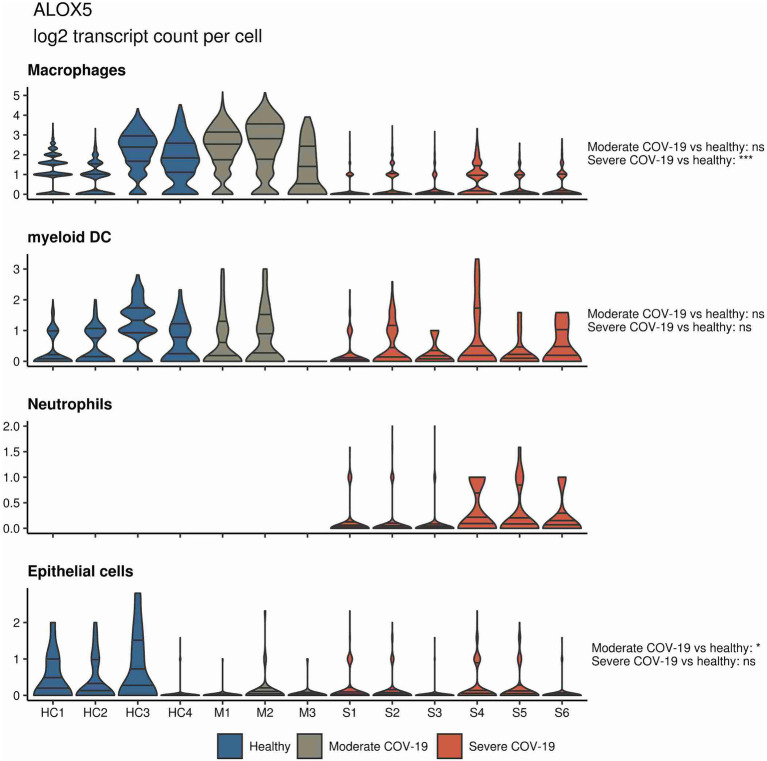
*ALOX5* expression in bronchoalveolar lavage fluid (BALF) cells from patients affected by COVID-19. Violin plots depicting *ALOX5* expression levels within specific cellular populations in BALF cells from healthy (blue, *n* = 4), moderate (gray, *n* = 3), or severe (red, *n* = 6) COVID-19 patients. RNA sequencing data are publicly available (Liao et al., 2020). ns = non-significant, ^***^p < 0.001.

**Figure 3:**
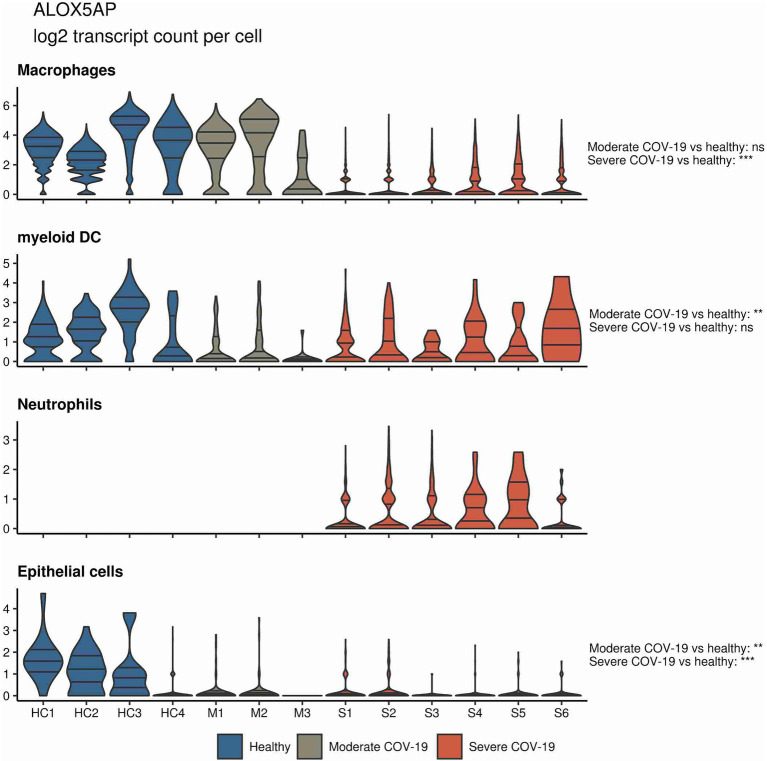
*ALOX5AP* expression in BALF cells from patients affected by COVID-19. Violin plots depicting *ALOX5AP* expression levels within specific cellular populations in BALF cells from healthy (blue, *n* = 4), moderate (gray, *n* = 3), or severe (red, *n* = 6) COVID-19 patients. RNA sequencing data are publicly available (Liao et al., 2020). ns = non-significant, ^**^p < 0.01, ^***^p < 0.001.

**Figure 4:**
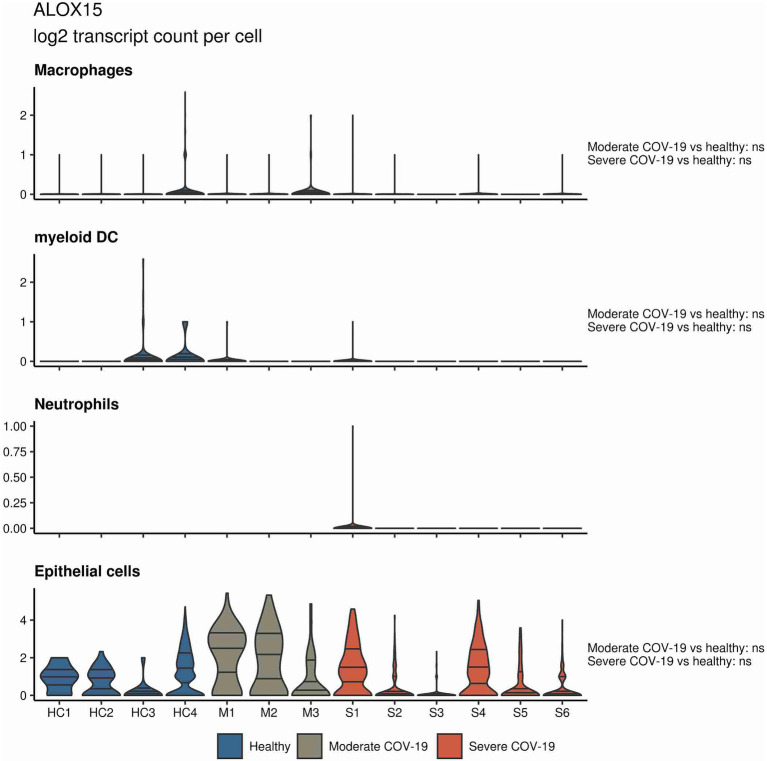
*ALOX15* expression in BALF cells from patients affected by COVID-19. Violin plots depicting *ALOX15* expression levels within specific cellular populations in BALF cells from healthy (blue, *n* = 4), moderate (gray, *n* = 3), or severe (red, *n* = 6) COVID-19 patients. RNA sequencing data are publicly available (Liao et al., 2020). ns = non-significant.

**Figure 5:**
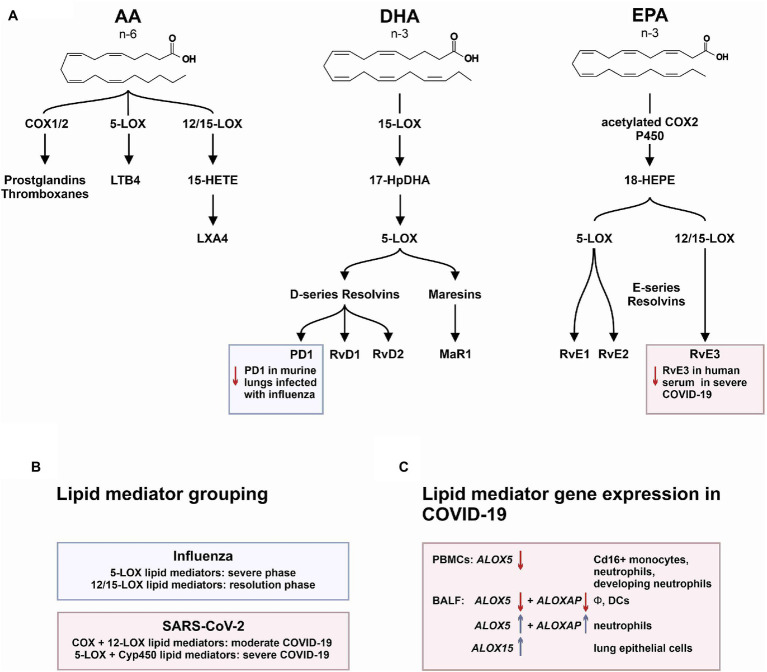
The mediator lipidome in influenza and SARS-CoV-2 infection. **(A)** The graph summarizes common 6-PUFA- and 3-PUFA-derived bioactive and pro-resolving lipid mediators (AA, arachidonic acid; DHA, docosahexaenoic acid; and EPA, eicosapentaenoic acid). While severe influenza infection showed inhibition of protectin D1 (PD1) synthesis, severe SARS-CoV-2 infection was associated with a significant reduction in E-series resolvin 3 (RvE3). **(B)** Lipid mediator grouping according to synthetic pathways. **(C)** Transcriptional regulation of key enzymes involved in lipid mediator synthesis in PBMCs and BALF cells from patients affected by COVID-19.

In a similar approach, our group interrogated a published single-cell RNA sequencing dataset from BALF in patients affected by COVID-19 (Liao et al., 2020) and found *ALOX5* to be downregulated in BALF macrophages and DCs from patients affected by severe COVID-19, compared to healthy individuals and to patients with moderate COVID-19. Vice versa, *ALOX5* expression was increased in BALF neutrophils in severe disease, although at an overall low expression level (Figure 2). 5-LOX requires a set of stimulatory factors for full activity and is supported by accessory proteins, including 5-LOX-activating protein (FLAP; *ALOX5AP*; Haeggström, 2018). Importantly, we found a decrease in *ALOX5AP* expression levels in macrophages and DCs in BALF from severe COVID-19 patients, whereas RNA expression of this central activating protein tended to be increased in neutrophils of patients affected by severe disease (Figure 3). Finally, the expression of a LOX involved in the synthesis of pro-resolving lipid mediators, namely, *ALOX15*, was found highest in lung epithelia of patients affected by moderate COVID-19, potentially conferring an anti-inflammatory role to this cellular lung compartment upon SARS-CoV-2 infection (Figure 4). These data indicate differences in expression and potentially activity of 5-LOX between pulmonary macrophages and circulating monocytes in patients with severe COVID-19. Moreover, the differences in expression of *ALOX5* and *ALOX5AP* in cells of myeloid origin found in the lungs of patients with moderate and severe disease may contribute to the specific differences in abundance and immune-modulatory functions of resolvins and lipoxins (Schwarz et al., 2021; An overview on the main lipid metabolome changes in influenza and SARS-CoV-2 infection is given in Figure 5).

Interestingly, LXA4 and isomers of the D-series resolvin RvD6 were previously found to reduce the expression of ACE2 and to counteract the binding of the receptor-binding domain of SARS-CoV-2 spike protein to injured tissue (Pham et al., 2020). The SARS-CoV-2 spike protein S1 subunit was moreover found to induce RvD1 in macrophages from patients affected by cystic fibrosis (CF). Importantly, RvD1 and RvD2 counteracted the inflammatory response to SARS-CoV-2 spike protein in both CF and non-CF macrophages, while potentiating their host defensive, phagocytic functions (Recchiuti et al., 2021).

From a clinical perspective, it will be important to develop biomarkers allowing to predict the course of a COVID-19 infection, to stratify patients for specific treatments and deduce novel potential targets to prevent a severe course of infection. Given the fact that LOX and FLAP proteins have been identified as key players in SARS-CoV-2 infections, several possibilities arise. With respect to disease and patient stratification biomarkers, LOX pathway markers, such as 5-HETE, 15-HETE, and other mono-hydroxylated PUFA derivatives, should be considered as possible diagnostic tools to predict the subsequent course of the infection and to induce specific targeted therapies. With regard to therapeutic interventions, omega-3 PUFA as investigated in the *COVID-Omega-F* Trial may represent an attractive approach to counteract pathologic inflammation thereby preventing lung dysfunction and need for mechanical ventilation. This view has just recently been backed up by Darwesh et al. proposing omega-3 PUFA as adjuvant therapy (Darwesh et al., 2021). Additionally, several drugs, such as for example montelukast or zileuton, have been designed to target leukotrienes in inflammatory lung diseases. As outlined by Funk and Ardakani (2020), a dual-treatment paradigm targeting leukotrienes as the final pro-inflammatory mediators of the 5-LOX pathway might pose a scientifically sound approach, which to our knowledge has however been neglected so far. Unfortunately, this counts for the entire eicosanoid pathway as recently outlined by Hammock et al. (2020), even though it bears a central role in pro- and anti-inflammatory responses triggered by infectious agents (Dennis and Norris, 2015). In fact, the eicosanoid pathway presents several interesting targets for the treatment of COVID-19. In addition to the targets suggested by Funk and Ardakani, COX, microsomal prostaglandin E2 synthase-1 (mPGES-1; Bergqvist et al., 2020) as well as soluble epoxide hydrolase inhibitors (Hammock et al., 2020) present interesting novel avenues for the treatment of COVID-19. Particularly, the latter might ideally be combined with omega-3 PUFA substitution, boosting the production of epoxyeicosanoids exerting anti-inflammatory as well as tissue regenerative functions (Morisseau and Hammock, 2013).

Besides the extensive research on SARS-CoV-2 and the gained knowledge, there are still points that require further investigation to complete the picture of SARS-CoV-2 infection, COVID-19 disease progression and resolution. Increasing the knowledge about the complex interplay between lipid mediators, the immune system and SARS-CoV-2 infection will yield novel insights into underlying pathomechanisms and will provide the basis for novel therapeutic strategies.

## Materials and Methods

Single-cell sequencing data provided by Liao et al. (2020) were analyzed with R programing suite version 4.0.3 and tidyverse package bundle. In brief, transcript counts per cell in BALF macrophages from healthy and COVID-19 individuals were extracted from the table with normalized expression and sample-cell-individual assignment table provided by the authors. For visualization as violin plots (package ggplot2), transcript counts were transformed with the log2(x + 1) function. Statistical significance for differences in transcript numbers per cell between the COVID-19 severity groups and healthy controls was assessed by mixed-effects generalized linear modeling (log link function, expected distribution of residuals: Poisson, package lme4) with the fixed effect of the study group and the random effect of the cell donor.

## Author Contributions

SS, IT, RM, GW, AT, and MG wrote the manuscript. PT performed bioinformatics analyses. All authors critically reviewed the final version of the manuscript.

### Conflict of Interest

The authors declare that the research was conducted in the absence of any commercial or financial relationships that could be construed as a potential conflict of interest.
